# Implementation of a Digital Tool to Identify Child Development and Parental Mental Health Needs Among Multicultural Communities Attending a School‐Based Wellbeing Hub

**DOI:** 10.1111/cch.70321

**Published:** 2026-07-17

**Authors:** Ewan Shao‐Jin Mok, Christa Lam‐Cassettari, James Rufus John, Poppy Loueizi, Olivia Wright, Valsamma Eapen

**Affiliations:** ^1^ School of Clinical Medicine, Discipline of Psychiatry and Mental Health University of New South Wales Sydney NSW Australia; ^2^ Academic Unit of Infant, Child, and Adolescent Psychiatry Services (AUCS), SWSLHD Sydney NSW Australia; ^3^ Ingham Institute of Applied Medical Research Sydney NSW Australia; ^4^ Ashcroft Public School Sydney NSW Australia; ^5^ School Gateway Project, NCOSS ‐ Yirranma Place Darlinghurst NSW Australia

**Keywords:** child development, health inequity, school‐based hub, screening

## Abstract

**Background:**

Evidence supports developmental surveillance and integrated care hubs for early identification and intervention in child developmental vulnerabilities. However, data on their use in community settings including schools and multicultural populations is limited. This study examined the implementation of the Watch Me Grow‐Electronic (WMG‐E) developmental surveillance tool and staff perspectives at the Mirrung wellbeing hub integrated with a preschool in South‐Western Sydney.

**Methods:**

A mixed‐methods study recruited parents/carers of children aged 3–6 years enrolled at Ashcroft Public School to complete WMG‐E developmental screening assessments. Additionally, qualitative interviews with Mirrung staff assessed implementation metrics including acceptability, adoption, appropriateness, coverage and sustainability. Multilevel binary logistic regression models were conducted to determine whether sociodemographic and clinical characteristics were associated with the occurrence of developmental concern. Qualitative data was analysed thematically.

**Results:**

Lower parent/carer education was associated with three‐fold higher risk of child developmental concern (AOR 3.36, 95% CI 1.06–12.44). Thematic analysis revealed three themes: Barriers to Service Access, Enablers to Service Access and Uptake of WMG‐E at Mirrung.

**Conclusion:**

WMG‐E is a feasible, appropriate and acceptable developmental screening tool for multicultural school‐based settings. Findings emphasise the need for culturally relevant health literacy resources to improve parents/carers' engagement to support early child development.

## Summary

- Leveraging the relationship that preschool staff have with parents supports completion of developmental screening and identification of child healthcare needs in 4–6‐year‐old children.
- The Watch Me Grow‐Electronic (WMG‐E) digital screening tool supported families to complete developmental checks in their own time.
- Multicultural school settings were identified as locations that would benefit from increased health literacy on developmental screening and access to health services that support milestone development.

## 1 Introduction

Developmental vulnerability is defined as challenges in any of five domains: physical health and wellbeing, social competence, emotional maturity, school‐based language and cognitive skills, or communication and general knowledge (scores below 10th percentile) (Department of Education, Skills and Employment 2021). Developmental vulnerabilities can adversely impact the life trajectory outcomes for children, resulting in significant health inequities and poorer physical health, mental wellbeing and psychosocial outcomes (World Health Organization 2023; Berg et al. 2019). In Australia, approximately 22% of children are developmentally vulnerable in one or more developmental domains at the start of school (Department of Education, Skills and Employment 2021). Early intervention has been shown to greatly support child developmental outcomes and life functioning (Rinaldi et al. 2021; Fuller and Kaiser 2020; Shaw et al. 2012). However, access to intervention is dependent on the early identification of developmental vulnerabilities (Garg et al. 2017). Developmental surveillance is defined as a ‘flexible, continuous process whereby knowledgeable professionals perform skilled observations of children during the provision of health care’ and is critical for early identification (Dworkin 1993). Despite the beneficial outcomes of early identification of developmental vulnerabilities, the uptake of developmental surveillance is critically low in Australia, particularly among priority populations such as Indigenous and culturally and linguistically diverse children, and declines as children age (Eapen et al. 2017; Woolfenden, Eapen, Axelsson, et al. 2016; Eapen et al. 2022). Children from disadvantaged families experience an ‘inverse care law’ in that they are at higher developmental risk but are least likely to engage in prevention and health promotion programs such as developmental surveillance. Further, parents/carers from culturally and linguistically diverse (CALD) backgrounds face additional barriers to accessing services due to factors including limited English proficiency, lower parental education, lower socioeconomic status, cultural beliefs and awareness of available services, to name a few (Garg et al. 2017).

To address disparities in the uptake of developmental surveillance and intervention among CALD children in vulnerable regions, implementing a digital developmental surveillance tool has been found to be beneficial (Barr et al. 2024). With advancements in information technology and internet accessibility, innovative eHealth approaches such as parent‐reported screening tools and algorithm‐based risk assessments may enhance developmental surveillance (Maleka et al. 2016; Smart 2018; Brooks et al. 2016; Kohlhoff et al. 2022). Existing research suggests that digital developmental screening/surveillance tools are acceptable and effective and in particular feasible and beneficial for reaching underserved populations, thereby increasing screening rates, supporting clinician's decision‐making and enhancing parent/carer health literacy on child development (Brooks et al. 2016; Kohlhoff et al. 2022; Baker et al. 2020). Further, when used by child health workers, digital screening tools may produce outcomes comparable to gold‐standard tools used by health professionals (Maleka et al. 2016).

### 1.1 Place‐Based Approaches (PBAs)

Despite the potential of digital developmental surveillance tools, their uptake also remains limited when access is confined to traditional healthcare settings, particularly in more vulnerable regions with large CALD populations (Garg et al. 2017; Ayer et al. 2020; Overs et al. 2017; Garg et al. 2018). Place‐based approaches (PBAs) respond to challenges within specific geographic areas and have been used to provide services to underserved communities to improve child developmental outcomes as well as parental (e.g., employment, education), family and community (e.g., accessible services, interconnectedness) factors that influence child developmental trajectories (Burgemeister et al. 2021; Moore et al. 2014). PBAs typically involve collaboration between community led organisations, professionals, and the local community, and leverage local knowledge to ensure programs are contextually appropriate and effective (Butler et al. 2022; Taylor et al. 2017; Slattery et al. 2020). For example, co‐designed child and family centres have been perceived as welcoming and supportive, helping parents build positive connections and facilitating their engagement in early childhood services (Taylor et al. 2017). Utilising opportunistic contacts alongside PBAs could overcome some of the local barriers including inequity in healthcare access which in turn can improve both child outcomes and community support (Burgemeister et al. 2021).Given the success of co‐designed PBAs in tailoring services to local needs, strategically located integrated child and family hubs can effectively address specific challenges faced by children and parents (Montgomery et al. 2024).

### 1.2 Well Being Hub (Mirrung)

School‐based hubs as PBAs offer a potential opportunity whereby families, educators, healthcare professionals and community members may converge and partner to promote the healthy development of children (Jacobson 2016). Children's developmental concerns are often first identified by teachers and schools, demonstrating their potential value in aiding developmental surveillance ('Connor et al. 2019; 'Connor et al. 2020). However, families with limited health literacy may inadequately communicate developmental concerns and teachers may encounter barriers in engaging parents and addressing these issues (Morrison et al. 2019; Nash et al. 2020). School hubs are thus beneficial in bridging communication gaps, facilitating engagement with assessments and interventions, and offering accessible pathways for developmental surveillance and service access (Jacobson 2016; 'Connor et al. 2019; Edwards et al. 2020). For example, an Australian school‐based health hub ‘Our Mia Mia (OMM)’ has demonstrated effectiveness and acceptability in building stakeholder trust, streamlining parental help‐seeking, and facilitating coordinated, timely support (Mendoza Diaz et al. 2021; Burman et al. 2023).

The Mirrung Well Being Hub is a co‐designed PBA in South‐Western Sydney, in the state of New South Wales (NSW). The Hub location is characterised by a significant CALD population (59.1% non‐English speakers at home), highest poverty rate (32.9%) in the state and high rates of vulnerability compared to state average in areas such as employment, disability and access to health and developmental services (NSW Government 2023; Vidyattama et al. 2023; Australian Bureau of Statistics 2021). Mirrung—meaning ‘belonging’ in the Aboriginal Dharug language—is a cross‐sector initiative established through the partnership between the NSW Council of Social Services and Ashcroft Public School (APS). The Mirrung hub aims to improve student and family health and wellbeing by providing coordinated onsite services, fostering diverse partnerships, improving service access for families and supporting staff in identifying and addressing student needs.

### 1.3 Aims

Various studies have investigated the feasibility of a digital developmental surveillance tool in different community and healthcare settings (Eapen et al. 2017; Ayer et al. 2020). However, the use of digital developmental surveillance tools in school‐based hubs remains limited. To address this evidence gap, we aimed to examine the implementation and use of the Watch Me Grow‐Electronic (WMG‐E) digital developmental screening tool at Mirrung, informed by an implementation science perspective. Specifically, we sought to (i) describe the characteristics of parents/carers and their children and examine associations between sociodemographic factors, parental mental health and child developmental concerns and (ii) explore the perspectives of the staff about the implementation and any barriers in accessing child and family services, service provision and the uptake of the WMG‐E at Mirrung.

## 2 Methodology

### 2.1 Study Design

**FIGURE 1:**
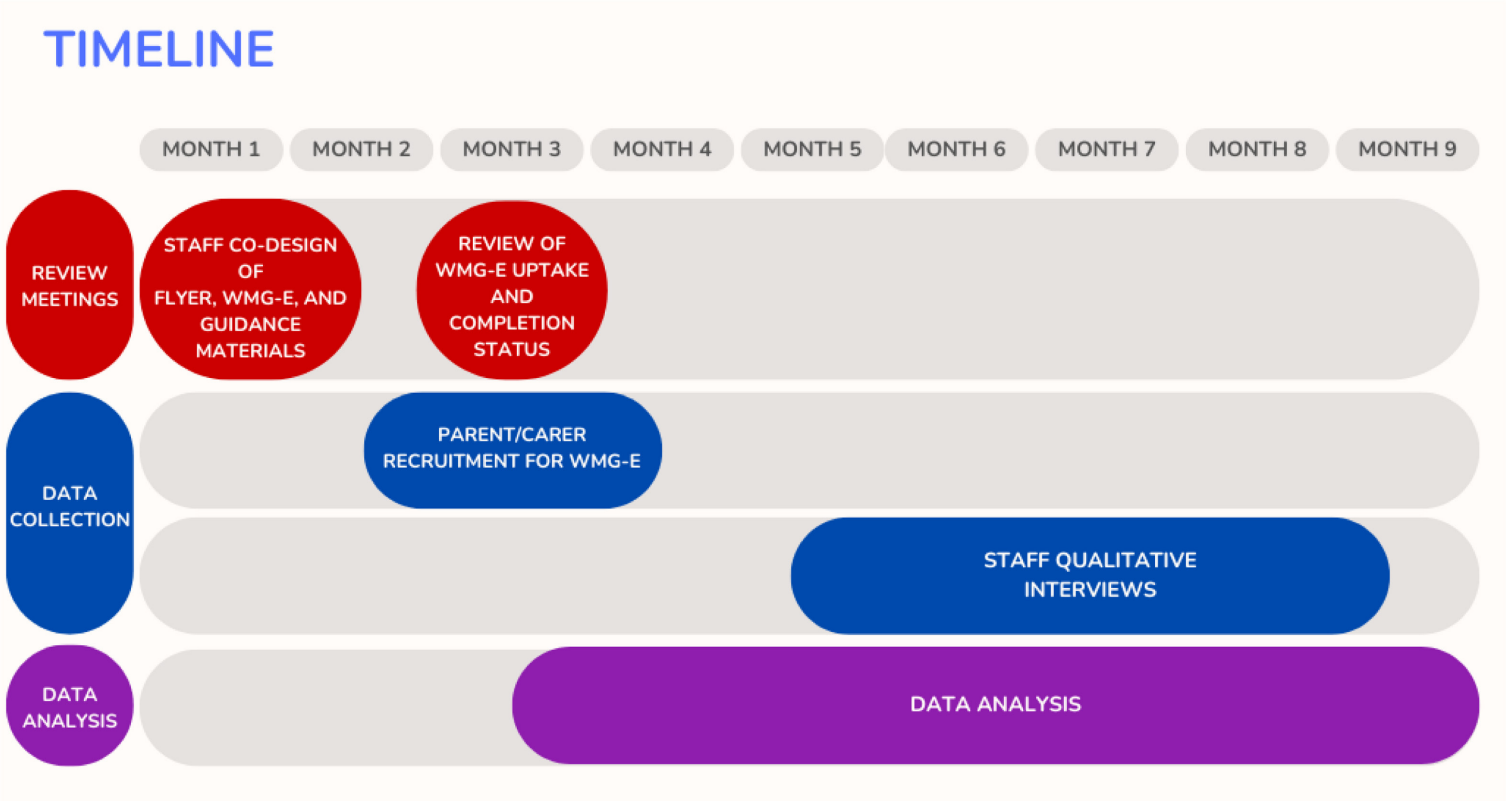
Data collection timeline.

This research utilised a mixed methods approach, incorporating both quantitative and qualitative methods. Sociodemographic information, quantitative measures of child developmental needs and parent/carer mental health were collected using parent‐report surveys via the WMG‐E tool. Additionally, semi‐structured interviews with Mirrung staff were employed to obtain a deeper understanding of their perspectives on the implementation of the wellbeing hub, service provision and the uptake of WMG‐E (Figure 1 illustrates the study timeline).

### 2.2 Participants—Eligibility Criteria, Recruitment and Consenting Process

All parents/carers of children aged 3–6 years were invited to complete the WMG‐E developmental screening tool by Mirrung staff via the distribution of flyers and verbal explanations on how to use a QR code to complete WMG‐E onlin*e*. Those who signed up via the QR code were subsequently recruited. Parents/carers provided online consent before accessing and completing the WMG‐E checks with staff support provided where required. Hub staff, including teachers, facilitators and senior administrative staff, were invited to participate in semi‐structured interviews once parent/carer participation was complete. Staff interviews were conducted after informed consent was provided.

Following completion of WMG‐E, hub staff reviewed each child's developmental profile with parents/carers, provided advice regarding relevant hub services, including developmental stimulation and support delivered by allied health staff, and facilitated referrals through the hub paediatrician and associated services when developmental vulnerabilities were identified. A structured protocol was also implemented for parents/carers reporting high psychological distress, whereby cases were escalated via the NSW Health clinical pathway to the South West Sydney Local Health District mental health team, referrals to local services were arranged as needed, and families were provided with anticipatory guidance and standardised support resources in the interim.

### 2.3 Data Collection

Data collection was conducted using the WMG‐E platform, an innovative digital developmental surveillance tool that aims to engage children and families by going to where they go (e.g., opportunistic service attendance such as immunisation visits, playgroups, early childhood education, and social services contacts) and once developmental needs are identified, they are provided tiered care based on their needs (Woolfenden et al. 2014).

#### 2.3.1 Child Developmental Outcomes

The WMG‐E tool comprises three parent‐report sections: (1) Centres for Disease Control ‘Learn the Signs Act Early’ (LTSAE) checklist to assess child developmental milestone progression, (2) Survey of Wellbeing of Young Children (SWYC) to assess preschool children's developmental and socio‐emotional functioning and (3) Kessler Psychological Distress Scale (K10) to assess parent/carer mental health (Centers for Disease Control and Prevention 2023; Eapen et al. 2021; Council on Children With Disabilities et al. 2006; Kessler et al. 2002). The LTSAE milestone checklist (12–14 items for 3–6 year olds) was used to identify ‘red flags’ in social/emotional, cognitive, language/communication and physical/movement domains (Raspa et al. 2015). If a parent/carer reports one or more ‘red flags’ on the LTSAE, an ‘at‐risk’ status is indicated, and the in‐built algorithm directs the participant to complete the SWYC. A higher LTSAE score indicates increased developmental vulnerability. The SWYC's 10‐item Developmental Milestones checklist (DM) and 18‐item Preschool Paediatric Symptom Checklist (PPSC) were used to identify any concerns in developmental and emotional/behavioural domains respectively (Tufts Medical Center 2023; Perrin et al. 2016). A higher DM score indicates a greater likelihood of developmental problems in the child. A higher PPSC score indicates a greater likelihood of emotional and behavioural problems in the child. If either the DM's or PPSC's specific score threshold is met, an ‘at risk’ status is indicated for that checklist. The outcome measure of ‘child developmental concern’ was determined if the PPSC and/or DM determined an ‘at risk’ status, dichotomised as 0 = no flag and 1 = flag.

#### 2.3.2 Parental Wellbeing

The Kessler's Psychological Distress Scale (K10) is a 10‐item questionnaire measured on a five‐point Likert scale to assess parent/carer psychological distress (Kessler et al. 2002). A higher K10 score indicates an increased likelihood of the participant having psychological distress.

#### 2.3.3 Sociodemographic Covariates

Sociodemographic information included age of child (in months), child's sex (male, female), language spoken at home (English, other), ethnicity (Australian, other), CALD background (yes, no), primary carer's education (post‐school qualifications, up to Year 12), primary carer's employment status (employed, unemployed), and estimated family income (≥$50 000, <$50 000).

All the above questionnaires were made available in the four most common languages spoken in south‐west Sydney: English, Vietnamese, Arabic and simplified Chinese.

#### 2.3.4 Semi‐Structured Interviews

All qualitative analyses were conducted online by XX using a semi‐structured interview guide. The interview guide included open‐ended questions exploring the social needs and experiences within the APS community in accessing child developmental services, and other service provision at Mirrung, and the use of WMG‐E as a developmental screening tool.

#### 2.3.5 Data Collection Timeline

### 2.4 Data Analysis

#### 2.4.1 Statistical Analyses

To address the first aim, descriptive analyses of baseline characteristics of parents/carers and their children were conducted and reported as means and standard deviations for continuous variables and as counts and percentages for categorical variables. In addition, exploratory analyses examined differences in baseline sociodemographic characteristics (e.g., child sex, parental educational status) and clinical indicators (e.g., presence of developmental concern), using independent samples *t*‐tests for continuous variables and chi‐squared tests for categorical variables. Differences in the baseline sociodemographic characteristics (e.g., gender, parental educational status) and clinical indicators (e.g., presence of child developmental concern) were assessed using independent sample *t*‐tests for continuous variables and Chi‐squared tests for categorical variables. If chi‐square test assumptions were violated, Fisher's exact test or likelihood ratio test were used as applicable. Next, underlying assumptions of the multilevel binary logistic regression models were tested, including correlation analyses using Pearson's correlation test between the sociodemographic predictors for multicollinearity. Finally, two multilevel binary logistic regression models were conducted to examine the relationship between sociodemographic factors, parental mental health, and child developmental concern (outcome variable). Associations were modelled as sociodemographic indicators only (Model 1) and parental mental health adjusted for sociodemographic indicators (Model 2). The significance level was set at 0.05 and all statistical tests were two‐sided. All analyses were undertaken in Statistical Package for the Social Sciences (SPSS) version 27 and RStudio version 2024.04.2+764.pro1 (IBM Corp 2020; RStudio, PBC 2020).

#### 2.4.2 Semi‐Structured Interviews

To address the second aim, a reflexive thematic analysis approach based on Braun and Clarke was used to develop themes and subthemes (Braun and Clarke 2006). De‐identified transcripts were created using Microsoft Word. Qualitative coding and thematic analysis were conducted using Taguette software (Rampin et al. 2021). Two researchers (XX and XX) independently reviewed transcripts for familiarisation, generating preliminary codes and themes. Thematic analysis was conducted iteratively, with codes and themes refined through discussion and with the research team.

## 3 Results

### 3.1 WMG‐E Survey

#### 3.1.1 Characteristics of the Participants

The sample comprised 61 of 73 parents/carers (83.6%) of children aged 3–6 years who completed the WMG‐E digital developmental screening tool. The baseline sociodemographic characteristics are shown in Table 1. Of the total sample of 61 parents/carers of children, 26 (42.6%) parents/carers had a child flagged for developmental concern while 35 (57.4%) parents/carers did not. The mean (±SD) age of the child at the time of data collection was 59.42 (±7.98) months with no significant child sex predilection (49.2% female, 50.8% male). A substantial proportion of parents/carers (80%) came from a CALD background based on language spoken at home or ethnicity. The mean parent/carer K10 score was 17.47 (±6.81 SD), which corresponds to moderate levels of psychological distress.

**TABLE 1:** Baseline sociodemographic characteristics of the sample.

Baseline sociodemographic characteristics	Total (*N* = 61)	No flag (*n* = 35)	Flag (*n* = 26)	*p*
Age of child at enrolment in months, mean (SD)	59.42 (7.98)	59.87 (8.55)	58.8 (7.26)	0.613
Child's sex				0.149
*Female*	30 (49.2%)	20 (57.1%)	10 (38.5%)	
*Male*	31 (50.8%)	15 (42.9%)	16 (61.5%)	
Language spoken at home				0.366
*English*	48 (78.7%)	26 (74.3%)	22 (84.6%)	
*Other*	13 (21.3%)	9 (25.7%)	4 (15.4%)	
Ethnicity				
*Australian*	12 (19.7%)	8 (22.9%)	4 (15.4%)	0.745
*Other*	48 (78.7%)	27 (77.1%)	21 (80.8%)	
*Missing*	1 (1.64%)	0 (0%)	1 (3.8%)	
CALD background^a^				0.745
*No*	12 (20.0%)	8 (22.9%)	4 (15.4%)	
*Yes*	48 (80.0%)	27 (77.1%)	21 (80.8%)	
Participant's education				0.055
*Post‐school qualifications*	21 (34.4%)	16 (45.7%)	5 (19.2%)	
*Up to year 12*	40 (65.6%)	19 (54.3%)	21 (80.8%)	
Participant's employment status				0.179
*Employed*	8 (13.1%)	6 (17.1%)	2 (7.7%)	
*Unemployed*	48 (78.7%)	24 (68.6%)	24 (92.3%)	
*Missing*	5 (8.20%)	5 (14.3%)	0 (0%)	
Estimated family income				0.240
*50 000 or more*	17 (27.9%)	12 (34.3%)	5 (19.2%)	
*Less than 50 000 per year*	38 (62.3%)	19 (54.3%)	19 (73.1%)	
*Missing*	6 (9.84%)	4 (11.4%)	2 (7.7%)	
Completed child's health check in Blue Book				0.746
*No*	12 (19.7%)	6 (17.1%)	6 (23.1%)	
*Yes*	49 (80.3%)	29 (82.9%)	20 (76.9%)	
Parent/caregiver psychological distress–K10 score, mean (SD)	17.47 (6.81)	17.43 (7.64)	17.52 (5.82)	0.963

*Note:* *p* Value from chi‐squared or Fisher's exact or likelihood ratio tests.

^a^ CALD status was computed using a combination of main language spoken at home (other than English) and/or ethnicity (other than Caucasian).

#### 3.1.2 Differences in Clinical Indicators by Child's Sex

A summary of the scores on clinical indicators is detailed in Table 2. While there were no significant differences between male and female children in SWYC DM score, SWYC PPSC score, and parental K10 score, a significant difference was found in the LTSAE scores where male children had significantly higher mean scores compared to female children (2.55 vs. 1.10).

**TABLE 2:** Difference in the clinical indicators by child's sex.

Clinical indicators	*N*	Overall	Male Mean (SD)	Female Mean (SD)	*p*
LTSAE score	61	1.84 (2.59)	2.55 (3.11)	1.10 (1.67)	0.027
SWYC DM score	33	11.18 (3.89)	10.90 (3.87)	11.62 (4.05)	0.614
SWYC PPSC score	33	11.09 (8.03)	11.20 (8.55)	10.92 (7.50)	0.925

*Note:* *p* Values calculated from independent samples *t*‐test based on child sex.

#### 3.1.3 Findings of the Binary Logistic Regression Analysis

Multilevel binary logistic regression models are presented in Table 3. Model 1 (sociodemographic indicators only) found that compared to a parent/carer with a higher education level (post‐school qualifications), parents/carers with a lower education level (up to year 12) had a 3.6 times higher risk of having a child with a developmental concern (AOR 3.63, 95% CI 1.06–12.44). Model 2 showed no significant associations between parental mental health and developmental vulnerability. Additionally, there were no significant interactions between any of the sociodemographic factors and parental mental health.

**TABLE 3:** Multilevel binary logistic regression model.

Variables	Model 1 AOR (95% CI)	Model 2 AOR (95% CI)
Age of child in months, mean (SD)	0.98 (0.90, 1.04)	0.98 (0.91, 1.05)
Child's sex		
*Female*	Ref category	Ref category
*Male*	2.06 (0.68, 6.22)	2.24 (0.71, 7.46)
CALD status^a^		
*No*	Ref category	Ref category
*Yes*	2.09 (0.52, 8.46)	2.12 (0.52, 9.90)
Primary carer's level of education		
*Post‐school qualifications*	Ref category	Ref category
*Up to year 12*	3.63 (1.06, 12.44)*	3.43 (1.02, 13.20)*
K10 score	—	1.43 (0.43, 4.86)

^a^ CALD status was computed using a combination of the main language spoken at home (other than English) and/or ethnicity (other than Caucasian).

* *p* value < 0.05.

### 3.2 Interview Findings

**FIGURE 2:**
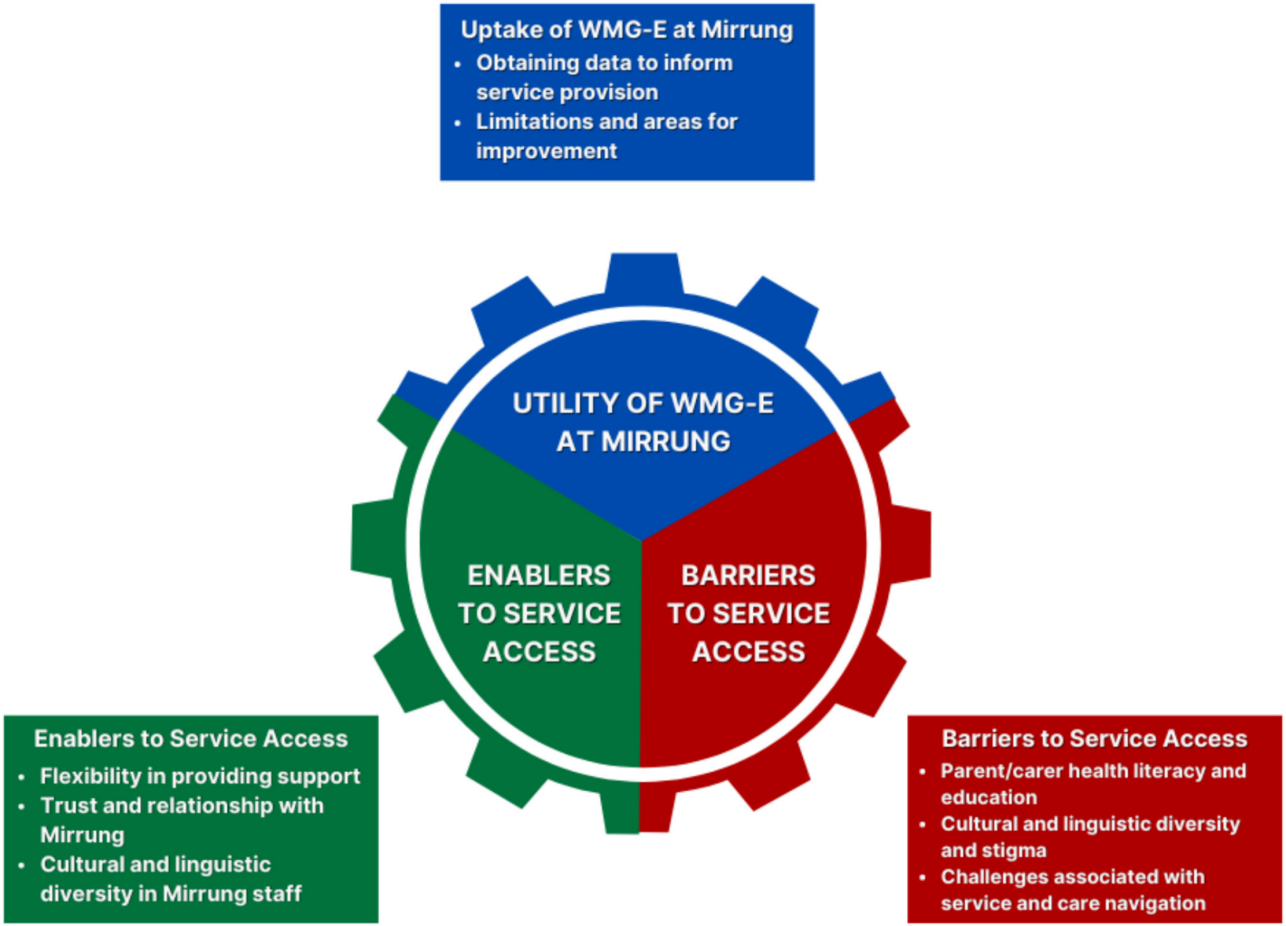
Conceptualisation of semi‐structured interview themes and subthemes.

Semi‐structured interviews were conducted with eight staff members (four executive and four teaching roles; mean age 42.5 years, SD 8.6; range 27–52). All had post‐school qualifications, and six were directly involved in administering the WMG‐E. Interviews lasted a mean of 33.5 min (SD 7.0; range 21–44). Three key themes were identified (Department of Education, Skills and Employment 2021) Barriers to Service Access, (World Health Organization 2023) Enablers to Service Access, and Uptake of WMG‐E. These themes reflect key implementation considerations relating to the PBA context and implementation processes. Themes and subthemes are summarised and conceptualised in Figure 2.

#### 3.2.1 Barriers to Service Access

Participants identified barriers to service access including (Department of Education, Skills and Employment 2021) parent/carer health literacy and education, (World Health Organization 2023) linguistic and cultural diversity and stigma associated with child developmental concern, and challenges associated with service and care navigation (Table 4).

**TABLE 4:** Barriers to service access.

Subthemes	Participant quotes
Parent/carer health literacy and education	Parents/carers lack ‘*education and awareness of what their children need to move forward*’ (P1) ‘*Child development information*’ is a significant issue with parents having a ‘*lack of developmental knowledge about their child*’ and that they ‘*do not know milestones*’ (P2) ‘Awareness’ and ‘Health information’ spreads ‘*far slower in a community like this that's very multicultural, low socioeconomic baseline, and low in parent education*’ (P5).
Cultural and linguistic diversity and stigma	‘*There are families who would not be confident to approach the school because of a language barrier or cultural barrier*’ (P4) ‘It's definitely a cultural barrier’ for a number of families ‘because they are all English‐speaking. It's not (just) a language barrier’ (P2) There is ‘*definitely a stigma attached to that (having children with developmental concerns) for a lot of families*’, making it challenging to have ‘*difficult conversations*’ (P2)
Challenges associated with service and care navigation	‘*The system is fragmented. It is hard to navigate. It is sometimes rigid, so the lack of flexibility in the services that we are trying to access is often difficult for families and therefore we have to navigate on their behalf*’ (P4) ‘*And the waiting list for rainbow cottage, which is the department in the hospital that sees the children can be anyway up to a year. So that's definitely a barrier*’, it is a ‘*systemic barrier*’ (P2)

Poor health literacy and lower levels of parent/carer education were identified as a significant barrier to accessing relevant services in the community (7/8 interviewees). Limited health literacy regarding the role of developmental screening in preschool‐aged children contributed to poor recognition of potential developmental concerns in their children and consequently limits the uptake of help seeking and appropriate early intervention. Participants also described barriers stemming from a lack of awareness and/or health literacy regarding the availability of community health services or information regarding expected child development milestones that resulted in parents missing out on opportunities to complete routine developmental screening.

All participants reported significant barriers surrounding culture, stigma, and language at various stages of accessing services. ‘There are families who would not be confident to approach the school because of a language barrier or cultural barrier’ (P4). There was also a reported cultural stigma that discourages parents/carers and cultural practices that served as barriers. ‘Navigating through medical services’ (P5) was reported to be difficult for ‘a lot of our families’ (P5) for whom ‘English is a second language’ (P5). Limited cultural sensitivity among health professionals and restricted support availability thus exacerbate challenges in accessing services.

Six participants identified challenges with service and care navigation as major barriers, noting a limited continuity of care. Long wait times were reported to be a significant contributor. Furthermore, navigating the health and support services system was difficult even for Mirrung staff, with it taking ‘a little bit to get going sometimes before you understand the benefits and the assistance that comes out of it’ (P1).

Financial strain and language barriers were identified as factors that compound other barriers to service access, contributing to the ‘cycle of disadvantage’ (P2).

#### 3.2.2 Enablers to Service Access

Participants identified that service access was facilitated by interrelated enablers, such as flexibility in providing support to parents/carers (Department of Education, Skills and Employment 2021), trust and relationship with Mirrung (World Health Organization 2023), and access to CALD staff (Table 5).

**TABLE 5:** Enablers to service access.

Subthemes	Participant quotes
Flexibility in providing support	‘Not being restricted into a formal process’ was ‘helpful’ ‘and so at this point the informal and flexible approach is what works best and whether or not that continues to be the best approach is yet to be seen’ (P4) ‘But in terms of the day‐to‐day things that parents sort of need to be referred for and things like that, if it's housing or food or whatever it is, then they are more sort of incidental and just those conversations with our staff’ (P3)
Trust and relationship with Mirrung	P3 stated that ‘*the biggest thing*’ is the ‘*the relationships and the trust*’ and that it is enabler if families ‘*can go to whoever they feel comfortable with*’ in the school. P4 when discussing the ideal referral process for Mirrung, states that ‘*You would speak to whomever in the school staff you had a relationship with, so whether that the teacher's aide, the classroom teacher or the front office or the executive, and you would give them a high level sense of what kind of support you needed, whether it was family support, health support, housing support*’ ‘*We're here if you need anything or if your child needs anything and we are just a phone call away*’ (P5)
Cultural and linguistic diversity in Mirrung staff	‘*A lot of our Arabic families really connect with one of our Arabic*’ staff because of ‘*cultural safety*’ and ‘*they know the customs*’ (P3) ‘*Employing an Aboriginal Education Officer has been a great step because she's actually having a lot more conversations with these parents that we were trying to engage*’ (P2)

Six participants described the current process at Mirrung of engaging and supporting or referring parents/carers as ‘informal’ (P1, P2, P5 and P6) and ‘flexible’ (P3 and P4)—and therefore a vital enabler to service access. Participants shared that while they have an ‘actual referral process in terms of learning’ (P3), there was also the flexibility of Mirrung to be ‘responsive and reactive’ (P5) to ‘urgent’ (P5) and ‘day‐to‐day things’ (P3) such as ‘housing or food’ (P3) or ‘grief counselling to the student’ (P5).

Seven participants described the trusting relationship between parents/carers and Mirrung as a critical enabler to service access and essential to Mirrung's model of care. ‘Talking to everybody’ (P2) was necessary as well as building relationships so that ‘our families already trust us’ (P3). Overall, all participants agreed that the ‘the biggest thing’ (P3) is ‘the relationships and the trust’ (P3).

Having staff who were from CALD backgrounds was also reported to be an enabler by five participants. The finding that staff from CALD backgrounds acted as an enabler complements the observation that cultural and linguistic diversity posed a barrier. P2 and P3 both reflected that, while difficult, more financial resources should be allocated towards hiring ‘more bilingual workers’ (P2) from CALD backgrounds to engage families from CALD backgrounds.

#### 3.2.3 Acceptability of WMG‐E at Mirrung

Participants' perspectives surrounding the acceptability of WMG‐E centred around (Department of Education, Skills and Employment 2021) obtaining data to inform service provision and (World Health Organization 2023) limitations and areas for improvement (Table 6).

**TABLE 6:** Uptake of WMG‐E at Mirrung.

Subthemes	Participant quotes
Obtaining data to inform service provision	P5 stated WMG‐E has ‘*given us some really rich data*’ to be the ‘*foundations*’ of the early intervention part of the (Mirrung) model. Using WMG‐E ‘*targeted and spotlighted the need and created an approach*’ to ‘*support kids with those developmental vulnerabilities*’ but must be seen ‘*as a tool as opposed to a solution to a problem*’ that ‘*is the beginning of the process*’ that helps to ‘*focus our attention*’ (P4) P3 stated that WMG‐E was helpful in initiating conversations with parent/carers ‘based on what you (the parent/carer) said about your child. So, I think it's been really helpful in terms of that’.
Limitations and areas for improvement	When asked if WMG‐E was easily adopted, P1 reflected: ‘*by the team, yes*’ but with ‘*parents, it was a little bit more difficult to implement*’ because ‘*it was difficult to get parents to come in and sit down with us*’ and ‘*quite a few families*’ ‘*did not have access to technology*’ or ‘*the confidence to actually complete the survey*’. When invited to sit with service providers to go through the survey, some families ‘*were not very receptive and were reluctant to come in*’. P2 stated that implementing WMG‐E was ‘*a bit clunky*’ and ‘*rushed*’

All participants advocated for the use of WMG‐E to complete developmental screening as a pivotal part of Mirrung's model of care. Participants valued the WMG‐E for its ability to obtain ‘really rich data’ (P5) which can be utilised to ‘focus our (Mirrung staff) attention’ (P4), ‘put some things in place’ (P3) in programs to accommodate different children accordingly, and initiate ‘those conversations’ (P3) with parents/carers about their children's developmental needs. P7 called the WMG‐E ‘highly beneficial’ as it ‘enables mass screening’, ensures ‘most issues are flagged early’, ‘helps parents navigate complex health and diagnostic systems’ and ‘provides a clear way to track students' progress’.

Participants reported that while WMG‐E was readily adopted by staff, uptake and implementation with families was difficult due to engagement barriers including limited ‘access to technology’ (P1), low ‘confidence to actually complete the survey’ (P1) and reluctance to attend in‐person support. P4 stressed the importance of WMG‐E to be seen ‘as a tool as opposed to a solution to a problem’ that is ‘the beginning of the process’ for families to engage in the developmental monitoring program, emphasising the tool's limited role.

## 4 Discussion

This study evaluated the initial implementation of WMG‐E in a PBA for identifying child developmental concerns and explored Mirrung staff's perspectives on service access inequity as well as their experiences in providing care to families within a multicultural community. As part of the exploratory analyses addressing the first study aim, WMG‐E survey results indicated that lower parental/carer education was a statistically significant predictor for child developmental concern. This is consistent with previous studies (Davis‐Kean 2005; Dubow et al. 2009; Moriguchi and Shinohara 2019; Waters et al. 2021; Tighe and Davis‐Kean 2021). For example, Moriguchi et al.’s study demonstrated that a parent's socioeconomic status, particularly education, predicts early childhood development in their children, with children of more educated and economically advantaged parents showing stronger vocabulary and better cognitive, social, and emotional development (Moriguchi and Shinohara 2019). Waters et al. (*N* = 1273) also found that a parent's education level was associated with their child's response inhibition, attention control, working memory and achievement (Waters et al. 2021). Such findings may be explained by the relationship between education and health literacy, which in turn influences parental health behaviour such as help‐seeking for unmet social or health needs including child developmental concern (Eapen et al. 2017; Cheng et al. 2016; Friis et al. 2016; van der Heide, Wang, et al. 2013; Bayati et al. 2018; Vamos and McDermott 2021). It is well established that educational level is associated with health literacy level as people with higher education levels have the necessary ability to find, read, understand and appraise health information (Friis et al. 2016; van der Heide, Wang, et al. 2013; Beauchamp et al. 2015; van der Heide, Rademakers, et al. 2013; Bo et al. 2014). In support, other quantitative studies conducted in South Western Sydney have found that lower parental educational levels predict non‐completion of developmental surveillance (Eapen et al. 2017; Ayer et al. 2020; Overs et al. 2017; Cuartas 2022). Health literacy has also been shown to mediate the association between educational attainment and health behaviour (Friis et al. 2016; van der Heide, Wang, et al. 2013; Bayati et al. 2018; Vamos and McDermott 2021). Hence, it is expected that by bringing developmental surveillance programs into preschools, hub staff can engage and empower parents by providing guidance particularly to parents with lower parental capacity, education and health literacy, thereby positively influencing health behaviours and reducing service access inequities. Further research is warranted to understand long‐term outcomes of this program.

As part of the reflexive thematic analysis conducted to address the second study aim, parent/carer health literacy and education were identified as key barriers to service access, consistent with other qualitative studies, including those involving CALD populations (Garg et al. 2017; Garg et al. 2018; Raspa et al. 2015; Ostojic et al. 2024; Woolfenden et al. 2015; Teng et al. 2007).

This finding also reflects the previous finding of an inverse care law, which posits that those who need healthcare the most tend to have the least access to quality care (Woolfenden et al. 2020). This disparity has been especially pronounced in CALD populations (Garg et al. 2017; Eapen et al. 2017; Woolfenden, Eapen, Axelsson, et al. 2016; Ayer et al. 2020; Overs et al. 2017). As identified by Mirrung staff, an intergenerational cycle of disadvantage is evident whereby lower parental/carer education contributes to developmental vulnerabilities and delays in their children, resulting in reduced educational outcomes and perpetuation of the cycle (Cheng et al. 2016). This however also highlights the value of education as an effective intervention to disrupt the intergenerational transmission of disadvantage (Andersen et al. 2021).

The identified subtheme of challenges associated with service and care navigation as a barrier to service access is also consistent with other studies (Burman et al. 2023; Ostojic et al. 2024; Woolfenden et al. 2015; Doucet et al. 2019; Breen et al. 2018). Notably, a study on OMM highlighted care navigation as a critical factor, emphasising their role of supporting parents as care navigators (Burman et al. 2023). The finding that cultural and linguistic diversity functions as an enabler when professionals are from CALD backgrounds and a barrier when it is not adequately addressed is consistent with other studies (Garg et al. 2017; Garg et al. 2018; Ostojic et al. 2024; Woolfenden et al. 2015). Cultural and linguistic diversity also compounds the issue of service and care navigation as reflected by participants and other studies (Garg et al. 2018; Ostojic et al. 2024; Teng et al. 2007). Therefore, it is crucial that screening tools, service providers and care systems incorporate culturally safe and sensitive accommodations.

The identified subtheme of trust and a relationship with service providers as an important enabler is consistent with other studies, particularly being highlighted in studies involving CALD families and hubs (Garg et al. 2017; Taylor et al. 2017; Burman et al. 2023; Ostojic et al. 2024; Woolfenden et al. 2015; Jose et al. 2019).This is significant as it indicates that Mirrung is well‐positioned to build trusting relationships with CALD families that facilitate developmental screening programs and service access. Similarly, flexibility was identified in other studies as a key enabler of service access and a barrier when absent, though participants in this study focused on its role as an enabler (Garg et al. 2018; Edwards et al. 2020; Burman et al. 2023). OMM's strength was notably reported to lie in its flexibility to adapt to meet population needs and integrate across care providers (Burman et al. 2023). Given that ‘flexibility’ is a key component in the definition of developmental screening, Mirrung may be well‐suited for implementing developmental screening and surveillance programs such as the WMG‐E in this context.

The Consolidated Framework for Implementation Research (CFIR) was applied as an interpretative lens to explore how the findings map onto CIFR domains (Reardon et al. 2025). Disparities in health literacy, complexity of service navigation and linguistic diversity were identified as barriers to the implementation of the WMG‐E screener in both inner and outer settings. In contrast, implementation enablers including trust, flexibility, and staff engagement were highlighted by the interviewees as supporting uptake of the WMG‐E screener and engagement with the hub. Together, these findings highlight the importance of understanding the local service hierarchies and processes so hub staff can adapt their approach when necessary to best support families in using a digital developmental screener in hub settings.

In their interviews, Mirrung staff highly valued WMG‐E as a tool to firstly, obtain developmental screening data and secondly, to inform practice and engage parents. This attitude towards developmental screening is also consistent with a previous WMG study where health professionals similarly valued screening tools for guiding themselves, parents, and initiating discussions (Garg et al. 2018). However, there are notable differences in reported experiences between Mirrung staff and the WMG study's health professionals. For example, one barrier faced by health professionals was poor continuity as they often do not see the same child regularly, limiting the effectiveness of one‐time assessments. In contrast, Mirrung staff see the same children for extended periods and can therefore utilise WMG‐E findings to design and tailor interventions accordingly. Another difference is that while Mirrung staff faced difficulty in engaging some parents to even begin utilising the WMG‐E, health professionals were able to leverage the opportunistic contact during consultations to utilise developmental screening tools. Although stakeholders' differing views stem from their professional contexts, these differences underscore the versatility and value of screening tools like WMG‐E across diverse settings, helping to address context‐specific challenges.

### 4.1 Strengths and Limitations

To our knowledge, this study is the first to provide evidence on the implementation of a digital developmental screening tool, which is critical to facilitate developmental screening in alternate community‐based settings such as preschools. Additionally, our findings offer diverse perspectives of Mirrung staff regarding enablers and barriers to service access and service provision in the context of a school‐based hub in a disadvantaged CALD community. Furthermore, the screening data collected from through the WMG‐E will facilitate future longitudinal comparisons at subsequent time points. However, this study has limited generalisability due to its small sample size and location in a defined, geographical region of Sydney with a population that is vulnerable, low‐income and largely from CALD backgrounds. Additionally, the relatively small sample size may reduce statistical power, resulting in imprecise estimates, as evidenced by the wide confidence intervals for some of the variables.

### 4.2 Implications

While the LTSAE is included as part of the NSW Health Personal Health Record (PHR or ‘Blue Book’) screening and surveillance program, its utilisation has been consistently low (Ayer et al. 2020; Garg et al. 2018; Ben‐Sasson et al. 2017; Woolfenden, Eapen, Jalaludin, et al. 2016). Findings of this study showed high uptake (61 out of 73 families; 83.6%) of the WMG‐E digital developmental screening tool enabling completion of child developmental checks and potential identification of any concerns that may be present. Additionally, WMG‐E was also highly recommended by hub staff to be feasible, acceptable and having immense value in increased coverage for identifying developmental concerns and informing practice within the preschool setting. Findings from Models 2–4 demonstrated the effectiveness of LTSAE and SWYC in identifying child developmental concerns. This emphasises the need to promote the use of digital developmental screening and surveillance tools such as WMG‐E and make it more accessible and flexible across health care systems over the currently used paper‐based version.

Identified barriers such as health literacy, parent education, and linguistic diversity as well as the relationship between parent/carer education and likelihood of child developmental concern suggest a heightened need to prioritise health awareness programs and targeted interventions for both children and adults to address and mitigate the intergenerational transmission of disadvantage (Nash et al. 2020; Andersen et al. 2021; Heckman et al. 2010; Wilson et al. 2011). There is also a need to streamline the navigation of government and health services, potentially supporting the need for an integrated outreach approach (Edwards et al. 2020; Burman et al. 2023; Ostojic et al. 2024). Increased support is also required to help families access necessary supports promptly, to enhance Mirrung's current efforts in this area. In addressing any barriers to care, it is crucial to sustain efforts and funding to engage families from CALD backgrounds in a culturally sensitive manner and, where possible, in their native language.

## 5 Conclusion

This study sheds light on the use of WMG‐E as an acceptable, appropriate and feasible tool in improving coverage in a sustainable way to engaging and empower families to complete developmental checks to facilitate early identification of developmental concerns in this multicultural community. Perceived barriers in current service provision included the need to raise awareness and increase health literacy around the importance of developmental screening among multicultural and relatively disadvantaged communities through culturally sensitive methods and supported by trusted providers such as preschools. Further research evaluating the effectiveness of using WMG‐E in PBA to improve longer‐term child developmental and parental mental health outcomes beyond the initial identification of concerns is required.

## Author Contributions

**Ewan Shao‐Jin Mok:** investigation, formal analysis, writing – original draft, writing – review and editing, project administration, visualization, data curation. **Christa Lam‐Cassettari:** conceptualization, methodology, data curation, investigation, formal analysis, supervision, project administration, writing – original draft, writing – review and editing, visualization. **James Rufus John:** conceptualization, methodology, formal analysis, supervision, writing – review and editing, project administration, visualization. **Poppy Loueizi:** conceptualization, writing – review and editing, resources, methodology. **Olivia Wright:** conceptualization, methodology, resources, writing – review and editing. **Valsamma Eapen:** conceptualization, methodology, supervision, resources, writing – review and editing.

## Funding

VE is supported by a Senior Research Leadership Fellowship (#2033610) from the National Health and Medical Research Council (NHMRC) of Australia.

## Ethics Statement

Ethics was approved by the SWSLHD Human Research and Ethics Committee (2023/ETH01374). Authorisation for the research was also granted through the NSW Department of Education's State Education Research and Partnerships (SERAP) application process (2023225).

## Conflict of Interests

The authors declare no conflicts of interest.

## Data Availability

Deidentified data will be made available upon reasonable request.
